# Systemic immune-inflammation Index is an independent risk factor for Major adverse cardiovascular events in patients with coronary artery ectasia

**DOI:** 10.3389/fcvm.2026.1839237

**Published:** 2026-07-09

**Authors:** Deguang Wang, Jingxian Xing, Zhaoqing Xie, Yunlong Zhang, Yunjie Wu, Tao Geng

**Affiliations:** Department of Coronary Heart Disease 7, Cangzhou Central Hospital, Cangzhou, China

**Keywords:** coronary artery ectasia, inflammation, major adverse cardiovascular events, risk stratification, systemic immune-inflammation index

## Abstract

**Background:**

Reliable biomarkers for long-term risk stratification in coronary artery ectasia (CAE) remain limited. The systemic immune-inflammation index (SII), derived from routine hematological parameters, reflects the balance between inflammation and immune status and has demonstrated prognostic value in various cardiovascular conditions.

**Objective:**

This study aimed to evaluate whether SII independently predicts major adverse cardiovascular events (MACE) in patients with angiographically confirmed CAE.

**Methods:**

In this retrospective cohort study at Cangzhou Central Hospital, 200 consecutive patients with CAE were enrolled and followed for a median duration of 30 months. SII was calculated as platelet count×neutrophil count divided by lymphocyte count. The primary endpoint was MACE, defined as a composite of cardiovascular death, nonfatal myocardial infarction, ischemic stroke, and target vessel revascularization. Receiver operating characteristic (ROC) curve analysis was performed to determine the optimal SII cut-off value. Kaplan–Meier survival analysis and Cox proportional hazards regression models were used to assess the association between SII and outcomes. Incremental predictive value was evaluated by comparing model discrimination and reclassification indices.

**Results:**

During follow-up, 18% of patients experienced MACE. Baseline SII levels were significantly higher in patients who developed adverse events. ROC analysis demonstrated good discriminatory ability of SII for predicting MACE (AUC 0.81), with an optimal cut-off value of 645. Kaplan–Meier analysis showed significantly lower event-free survival in patients with high SII levels. In multivariate Cox regression analysis, SII remained independently associated with MACE both as a continuous variable (adjusted HR 1.72 per SD increase) and as a categorical variable (adjusted HR 2.48 for high vs. low SII). Addition of SII to a baseline clinical model significantly improved discrimination (C-statistic increase from 0.72 to 0.83) and enhanced risk reclassification.

**Conclusion:**

Elevated systemic immune-inflammation index is an independent predictor of major adverse cardiovascular events in patients with coronary artery ectasia. Incorporation of SII into clinical risk assessment models significantly improves prognostic accuracy, suggesting that this readily available biomarker may serve as a valuable tool for risk stratification in this high-risk population.

## 1 Introduction

Coronary artery ectasia (CAE) is defined as a localized or diffuse dilation of a coronary artery segment measuring at least 1.5 times the diameter of the adjacent normal segment (1). Although often detected incidentally during coronary angiography, CAE represents a distinct coronary pathology rather than a simple anatomical variant. The reported prevalence ranges between 1% and 5% among patients undergoing coronary angiography, with atherosclerosis considered the most common underlying etiology (2).

The pathophysiology of CAE is complex and incompletely understood. Chronic vascular inflammation plays a central role in its development and progression. Histopathological studies demonstrate destruction of the medial layer, degradation of extracellular matrix components, and excessive remodeling of the arterial wall (3). Inflammatory cell infiltration, increased oxidative stress, and dysregulated matrix metalloproteinase activity contribute to weakening of the vessel wall and progressive dilation (4). Endothelial dysfunction further exacerbates this process by impairing vascular homeostasis and promoting a pro-inflammatory and pro-thrombotic microenvironment (5). In addition to structural abnormalities, altered coronary hemodynamics in ectatic vessels predispose patients to blood stasis, turbulent flow, and enhanced platelet activation. These changes increase the risk of intracoronary thrombus formation, distal embolization, and recurrent ischemic events (6). Consequently, patients with CAE may experience angina, acute coronary syndromes, or other major adverse cardiovascular events (MACE), even in the absence of significant obstructive coronary stenosis.

Despite its recognized clinical implications, risk stratification in CAE remains challenging. Traditional cardiovascular risk factors and anatomical classifications, such as the Markis classification, do not adequately predict long-term outcomes (7). Currently, there is no established biomarker specifically validated for prognostic assessment in CAE patients. Given the central role of inflammation and thrombosis in CAE pathogenesis, identifying simple and reliable inflammatory indices that can predict adverse outcomes may improve clinical management and follow-up strategies.

Inflammation plays a central role in the initiation, progression, and destabilization of atherosclerotic plaques. Accumulating evidence demonstrates that inflammatory activation not only contributes to plaque formation but also determines plaque vulnerability and subsequent thrombotic complications (8). Among circulating immune cells, neutrophils, platelets, and lymphocytes have emerged as key mediators linking inflammation to adverse cardiovascular outcomes. Neutrophils are among the earliest responders in vascular injury and acute coronary syndromes. They promote endothelial dysfunction, release reactive oxygen species, and contribute to plaque instability through proteolytic enzyme secretion (9). Elevated neutrophil counts have consistently been associated with increased mortality and recurrent ischemic events in patients with coronary artery disease (CAD) and acute coronary syndromes (ACS). Platelets, beyond their classical role in hemostasis, actively participate in inflammatory signaling. Activated platelets interact with leukocytes and endothelial cells, amplify thrombo-inflammatory pathways, and facilitate intracoronary thrombus formation (10). In contrast, lymphocytes are considered protective components of the adaptive immune response. Reduced lymphocyte counts may reflect impaired immune regulation and have been associated with poor cardiovascular prognosis (11).

Given the intertwined roles of inflammation and thrombosis, composite hematologic indices integrating these parameters have gained increasing attention. The Systemic Immune-Inflammation Index (SII), calculated as platelet count  ×  neutrophil count divided by lymphocyte count, provides a comprehensive assessment of the balance between pro-inflammatory, pro-thrombotic, and immune regulatory pathways (12). Unlike single inflammatory markers, SII simultaneously captures three biologically relevant components of cardiovascular pathophysiology.

Recent studies have demonstrated that elevated SII is independently associated with adverse outcomes in various cardiovascular settings. In patients undergoing percutaneous coronary intervention, high SII levels predict major adverse cardiovascular events (MACE) during follow-up (13). Similarly, in ST-segment elevation myocardial infarction (STEMI) and broader ACS populations, increased SII has been linked to higher rates of mortality, recurrent myocardial infarction, and heart failure, even after adjustment for traditional risk factors (14, 15). These findings suggest that SII may serve as a robust and readily available prognostic marker in inflammatory-driven coronary conditions.

However, despite growing evidence supporting its prognostic role in obstructive CAD and ACS, the predictive value of SII in patients with coronary artery ectasia has not been adequately investigated. Given the prominent inflammatory and thrombotic mechanisms underlying CAE, SII may offer additional insight into risk stratification in this unique population.

Although growing evidence supports the role of inflammation in the pathogenesis of coronary artery ectasia (CAE), the prognostic implications of inflammatory biomarkers in this population remain insufficiently defined. Several recent studies have demonstrated that the Systemic Immune-Inflammation Index (SII) is significantly elevated in patients with isolated CAE compared with individuals with normal coronary arteries and, in some reports, compared with patients with obstructive coronary artery disease (16–18). Moreover, SII has been associated with the anatomical severity of ectasia, including the number of ectatic vessels and higher Markis classification types, suggesting a link between systemic inflammation and structural vascular remodeling in CAE (18).

However, these investigations have primarily focused on the diagnostic value of SII for identifying the presence of CAE or assessing angiographic severity. They were largely retrospective, single-center analyses without long-term follow-up data. Importantly, none of these studies comprehensively evaluated whether SII independently predicts major adverse cardiovascular events (MACE) in patients with CAE after adjustment for conventional cardiovascular risk factors and clinical variables. Given that CAE is characterized by chronic vascular inflammation, endothelial dysfunction, and enhanced thrombotic susceptibility, patients remain at risk for recurrent ischemic events even in the absence of significant obstructive stenosis. Yet, clinicians currently lack validated inflammatory biomarkers to stratify long-term risk in this specific population. While SII has consistently been shown to predict MACE in patients with obstructive coronary artery disease and acute coronary syndromes (13–15), its prognostic value in CAE has not been adequately investigated. Therefore, there remains a critical gap in the literature regarding whether SII can serve as an independent predictor of adverse cardiovascular outcomes in patients with CAE. Addressing this gap may provide a simple, inexpensive, and clinically accessible tool for improving risk stratification and guiding follow-up strategies in this under-recognized coronary condition.

The aim of the present study was to investigate whether the systemic immune-inflammation index is independently associated with major adverse cardiovascular events in patients with angiographically confirmed coronary artery ectasia. Specifically, we sought to evaluate the discriminatory performance of SII, determine its optimal prognostic cut-off value, assess its association with long-term outcomes using survival and regression analyses, and examine whether incorporation of SII improves risk stratification beyond conventional clinical and inflammatory risk factors.

## 2 Materials and methods

### 2.1 Study design and patient population

This retrospective observational cohort study was conducted at Cangzhou Central Hospital to evaluate the prognostic significance of the systemic immune-inflammation index (SII) in patients with coronary artery ectasia (CAE). Consecutive patients who underwent diagnostic coronary angiography between August 2023 and October 2024 were screened for eligibility. Patients were included if they were 18 years of age or older and had angiographically confirmed CAE. Coronary artery ectasia was defined as a localized or diffuse dilation of a coronary artery segment measuring at least 1.5 times the diameter of the adjacent normal segment. The diagnosis was established through careful angiographic assessment by experienced interventional cardiologists.

To reduce potential confounding factors that might influence inflammatory markers, patients were excluded if they had significant obstructive coronary artery disease requiring urgent revascularization, acute or chronic infection at the time of evaluation, autoimmune or systemic inflammatory disorders, active malignancy, known hematological disease, severe hepatic or renal dysfunction, recent major surgery or trauma, or incomplete laboratory or follow-up data. Patients without adequate follow-up information were also excluded from the analysis. Cases with missing baseline laboratory measurements required for SII calculation or incomplete clinical records were excluded prior to analysis. Because all included patients had complete baseline and follow-up data, no imputation procedures for missing data were required. After applying these criteria, 200 patients were included in the final study population.

The study protocol was approved by the Ethics Committee of Cangzhou Central Hospital (No.:2024-257-02) and conducted in accordance with the ethical principles outlined in the Declaration of Helsinki. Due to the retrospective nature of the study and anonymized data collection, the requirement for written informed consent was waived.

### 2.2 Coronary angiography and definition of coronary artery ectasia

All coronary angiographic procedures were performed using standard radial or femoral access with the Judkins technique. Multiple angiographic projections were obtained to ensure complete visualization of the coronary arterial tree. All angiograms were independently reviewed by two experienced interventional cardiologists who were blinded to laboratory findings and clinical outcomes. In cases of disagreement, a consensus decision was reached. Coronary artery ectasia was defined as dilation of a coronary segment to at least 1.5 times the diameter of an adjacent normal segment. When no clearly normal adjacent segment was available, the reference diameter was estimated using the nearest angiographically normal vessel segment.

The anatomical severity of ectasia was categorized according to the Markis classification system. This classification system was used to quantify the extent of coronary dilation and was incorporated into subsequent statistical analyses to evaluate its association with adverse cardiovascular outcomes.

### 2.3 Clinical and laboratory data collection

Baseline demographic and clinical data were obtained from electronic medical records. Collected variables included age, sex, smoking status, history of hypertension, diabetes mellitus, and lipid profile parameters. Hypertension was defined as a documented history of elevated blood pressure or the use of antihypertensive medication. Diabetes mellitus was defined according to established diagnostic criteria or the use of glucose-lowering therapy. Smoking status was recorded as current smoker or non-smoker. Fasting venous blood samples were obtained at the time of hospital admission or during outpatient evaluation prior to any coronary intervention. Laboratory parameters included complete blood count components (white blood cell count, neutrophil count, lymphocyte count, monocyte count, platelet count, and hemoglobin level), lipid profile, and high-sensitivity C-reactive protein (CRP). Based on these hematological measurements, additional inflammatory indices including the neutrophil-to-lymphocyte ratio (NLR) and platelet-to-lymphocyte ratio (PLR) were calculated. All measurements were performed in the hospital's central laboratory using standardized automated analyzers with routine quality control procedures.

Transthoracic echocardiography was performed in all patients using a commercially available ultrasound system. Left ventricular ejection fraction (LVEF) was assessed using the modified Simpson's biplane method in accordance with current echocardiographic guidelines.

### 2.4 Calculation of systemic immune-inflammation Index

The systemic immune-inflammation index (SII) was calculated from baseline complete blood count parameters using the following formula:

SII=(Plateletcount×Neutrophilcount)/Lymphocytecount

All hematologic measurements were derived from the same baseline blood sample to ensure consistency. SII was analyzed as both a continuous variable and a categorical variable. For categorical analyses, an optimal cut-off value was determined using receiver operating characteristic curve analysis to identify the threshold that best discriminated between patients who did and did not experience major adverse cardiovascular events.

### 2.5 Study endpoint & follow-Up

The primary endpoint of the study was the occurrence of major adverse cardiovascular events (MACE). MACE was defined as a composite of cardiovascular death, nonfatal myocardial infarction, ischemic stroke, and target vessel revascularization. Cardiovascular death was defined as death attributable to myocardial infarction, heart failure, arrhythmia, stroke, or sudden cardiac death without a clearly identifiable non-cardiovascular cause. Myocardial infarction was diagnosed according to contemporary universal definition criteria. Ischemic stroke was confirmed by neurological evaluation and imaging studies. Target vessel revascularization included percutaneous coronary intervention or coronary artery bypass grafting involving the ecstatic vessel. Time-to-event was calculated from the date of index coronary angiography to the occurrence of the first MACE event or the last available follow-up.

Patients were followed through outpatient clinic visits, hospital record review, and structured telephone interviews conducted by trained investigators who were blinded to laboratory values. Follow-up data were successfully obtained for all patients included in the final analysis. Patients with unavailable follow-up information were excluded during the study selection process to ensure completeness of outcome assessment. Follow-up information included assessment of cardiovascular events, hospitalizations, and interventional procedures. For patients unable to attend in-person visits, telephone follow-up was performed using a standardized questionnaire. When necessary, additional documentation from referring physicians or external hospitals was obtained to verify reported clinical events. Patients who did not experience an endpoint event were censored at the time of their last clinical contact.

### 2.6 Statistical analysis

All statistical analyses were performed using SPSS statistical software (IBM Corp., Armonk, NY, USA) and MedCalc software. A two-sided *p*-value less than 0.05 was considered statistically significant. Continuous variables were assessed for normal distribution using the Kolmogorov–Smirnov test. Normally distributed variables were expressed as mean ± standard deviation, whereas non-normally distributed variables were presented as median with interquartile range. Categorical variables were expressed as frequencies and percentages. Comparisons between patients who developed MACE and those who did not were performed using the independent samples t-test or Mann–Whitney U test for continuous variables, and the chi-square test for categorical variables. The predictive performance of SII for MACE was evaluated using receiver operating characteristic curve analysis. The area under the curve was calculated to assess discrimination, and the optimal cut-off value was determined using the maximum Youden index. Event-free survival was analyzed using the Kaplan–Meier method, and differences between survival curves were assessed using the log-rank test.

Univariate Cox proportional hazards regression analysis was performed to evaluate the association between baseline demographic, clinical, laboratory, echocardiographic, and angiographic variables and the risk of MACE. Variables with a *p*-value < 0.10 in univariate analysis, together with clinically relevant covariates identified from previous literature, were entered into the multivariate Cox proportional hazards model to determine independent predictors. SII was evaluated both as a continuous variable per standard deviation increase and as a categorical variable based on the ROC-derived threshold. Hazard ratios with 95% confidence intervals were calculated. To evaluate incremental predictive value, the discrimination ability of a baseline model including conventional risk factors was compared with that of a model incorporating SII. Changes in the C-statistic were assessed. Net reclassification improvement and integrated discrimination improvement were calculated to quantify improvement in risk classification after inclusion of SII.

## 3 Results

### 3.1 Baseline characteristics

A total of 200 patients with angiographically confirmed coronary artery ectasia were included in the final analysis. The mean age of the study population was 58.6 ± 10.9 years, and 142 patients (71%) were male. The median follow-up duration was 30 months [interquartile range (IQR): 22–38 months]. During follow-up, 36 patients (18%) experienced major adverse cardiovascular events (MACE). The composite endpoint included cardiovascular death (*n* = 8), nonfatal myocardial infarction (*n* = 12), ischemic stroke (*n* = 5), and target vessel revascularization (*n* = 11). Patients who developed MACE were significantly older and had a higher prevalence of diabetes mellitus compared with those without events. However, after adjustment for other clinical and inflammatory variables, only diabetes mellitus remained an independent predictor of adverse outcomes. Baseline inflammatory markers were notably elevated in the MACE group. Median SII levels were significantly higher among patients who experienced MACE [812 (IQR: 645–1025)] compared with those who remained event-free [472 (IQR: 358–621), *p* < 0.001]. Similarly, CRP levels were significantly elevated in the MACE group. Furthermore, patients who developed MACE exhibited higher neutrophil-to-lymphocyte ratio (NLR) and platelet-to-lymphocyte ratio (PLR) values compared with event-free patients, indicating a more pronounced systemic inflammatory state. These findings support the contribution of inflammatory activation to adverse cardiovascular outcomes in patients with coronary artery ectasia. There were no significant differences between groups regarding sex distribution, hypertension, smoking status, or lipid profile.

Regarding ectasia severity, patients with MACE more frequently exhibited advanced Markis types (Type I–II) compared with the non-MACE group. Left ventricular ejection fraction (LVEF) was modestly but significantly lower in patients who experienced MACE. Baseline clinical, laboratory, and angiographic characteristics are summarized in Table 1.

**Table 1:** Baseline characteristics of the study population.

Variable	Overall (*n* = 200)	MACE (*n* = 36)	No MACE (*n* = 164)	*p*-value
Age (years)	58.6 ± 10.9	62.8 ± 9.7	57.6 ± 11.0	0.006
Male sex, *n* (%)	142 (71%)	27 (75%)	115 (70%)	0.54
Diabetes mellitus, *n* (%)	64 (32%)	18 (50%)	46 (28%)	0.01
Hypertension, *n* (%)	108 (54%)	22 (61%)	86 (52%)	0.33
Current smoker, *n* (%)	78 (39%)	16 (44%)	62 (38%)	0.52
LDL-C (mg/dL)	116 ± 32	121 ± 35	115 ± 31	0.29
HDL-C (mg/dL)	42 ± 9	40 ± 8	43 ± 9	0.08
LVEF (%)	53.8 ± 6.4	50.9 ± 6.8	54.5 ± 6.1	0.002
CRP (mg/L)	4.1 (2.3–6.5)	6.8 (4.2–9.7)	3.6 (2.0–5.2)	<0.001
Markis Type I–II, *n* (%)	74 (37%)	20 (56%)	54 (33%)	0.01
Markis Type III–IV, *n* (%)	126 (63%)	16 (44%)	110 (67%)	—
SII	528 (372–704)	812 (645–1025)	472 (358–621)	<0.001
Neutrophil count ( × 10⁹/L)	4.8 (3.9–5.9)	5.8 (4.9–6.8)	4.5 (3.7–5.5)	<0.001
Lymphocyte count (×10⁹/L)	1.9 (1.5–2.3)	1.5 (1.2–1.9)	2.0 (1.6–2.4)	<0.001
Platelet count (×10⁹/L)	238 ± 62	258 ± 68	233 ± 59	0.03
NLR	2.78 (2.01–3.91)	3.92 (2.98–5.16)	2.46 (1.88–3.45)	<0.001
PLR	132 (104–168)	172 (138–214)	121 (98–154)	<0.001

Values are presented as mean ± SD, median (IQR), or *n* (%).

### 3.2 SII levels and MACE occurrence

**Figure 1:**
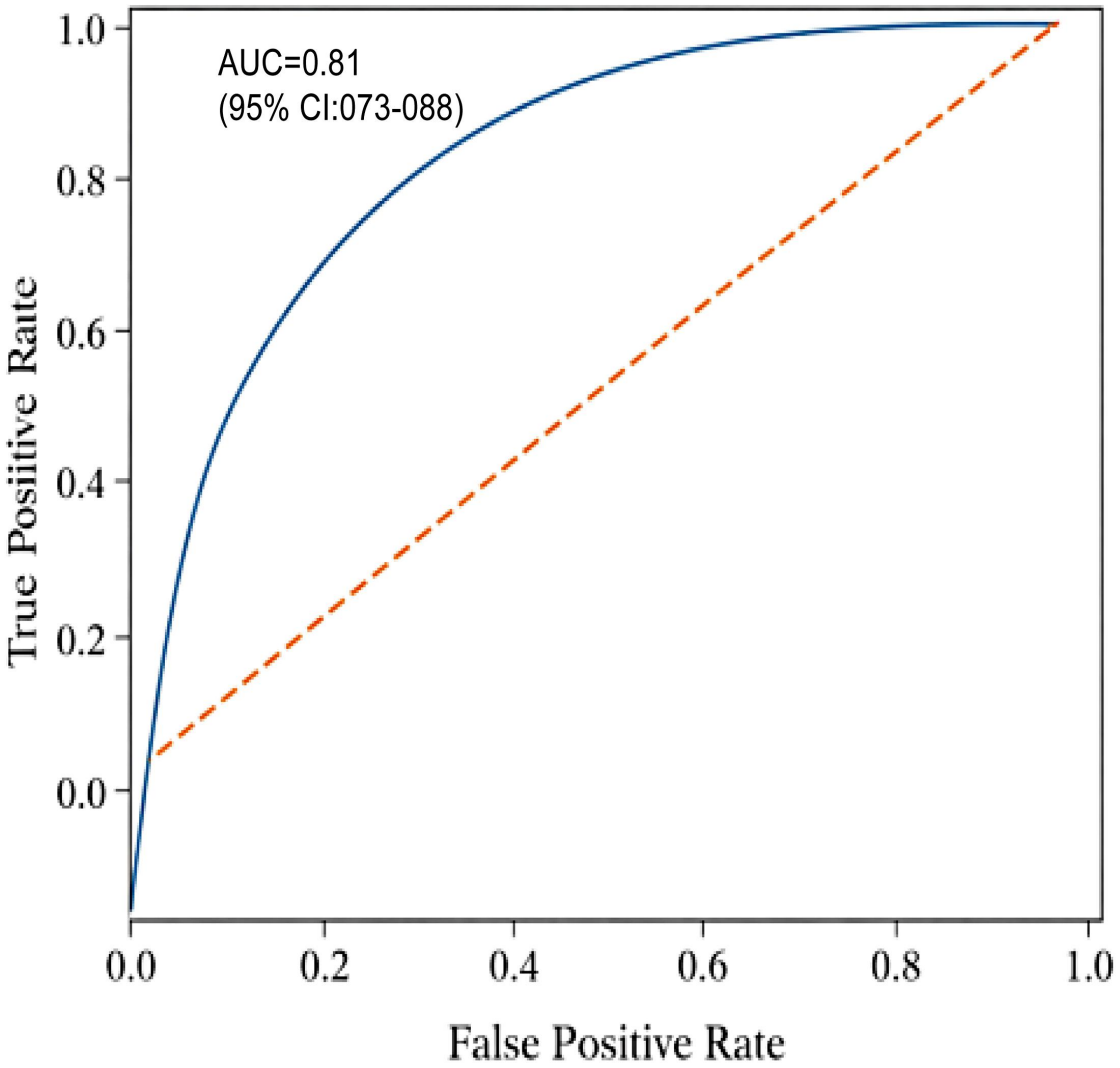
Receiver operating characteristic curve of systemic immune-inflammation Index for predicting Major adverse cardiovascular events. Receiver operating characteristic (ROC) curve illustrating the ability of the systemic immune-inflammation index (SII) to discriminate between patients with and without subsequent major adverse cardiovascular events (MACE) during follow-up. The area under the curve (AUC) was 0.81 [95% confidence interval (CI): 0.73–0.88, *p* < 0.001], indicating good predictive performance. The optimal cutoff value determined by the Youden index was 645, corresponding to a sensitivity of 78% and a specificity of 74%.

Systemic immune-inflammation index (SII) levels were significantly elevated in patients who developed major adverse cardiovascular events (MACE) during follow-up compared with those who remained event-free. The median SII value in the MACE group was 812 (IQR: 645–1025), whereas it was 472 (IQR: 358–621) in the non-MACE group (*p* < 0.001), indicating a strong association between heightened systemic inflammatory status and adverse cardiovascular outcomes in patients with coronary artery ectasia. To evaluate the predictive performance of SII for MACE, receiver operating characteristic (ROC) curve analysis was performed. As shown in Figure 1, SII demonstrated good discriminatory ability, with an area under the curve (AUC) of 0.81 (95% CI: 0.73–0.88, *p* < 0.001). The optimal cut-off value determined by the maximum Youden index was 645. At this threshold, SII predicted MACE with a sensitivity of 78% and a specificity of 74%. The positive predictive value was 41%, while the negative predictive value was 93%.

These findings suggest that elevated SII is strongly associated with adverse cardiovascular events in patients with CAE and demonstrates good discriminatory performance for identifying patients at increased risk of future MACE.

### 3.3 Survival analysis

**Figure 2:**
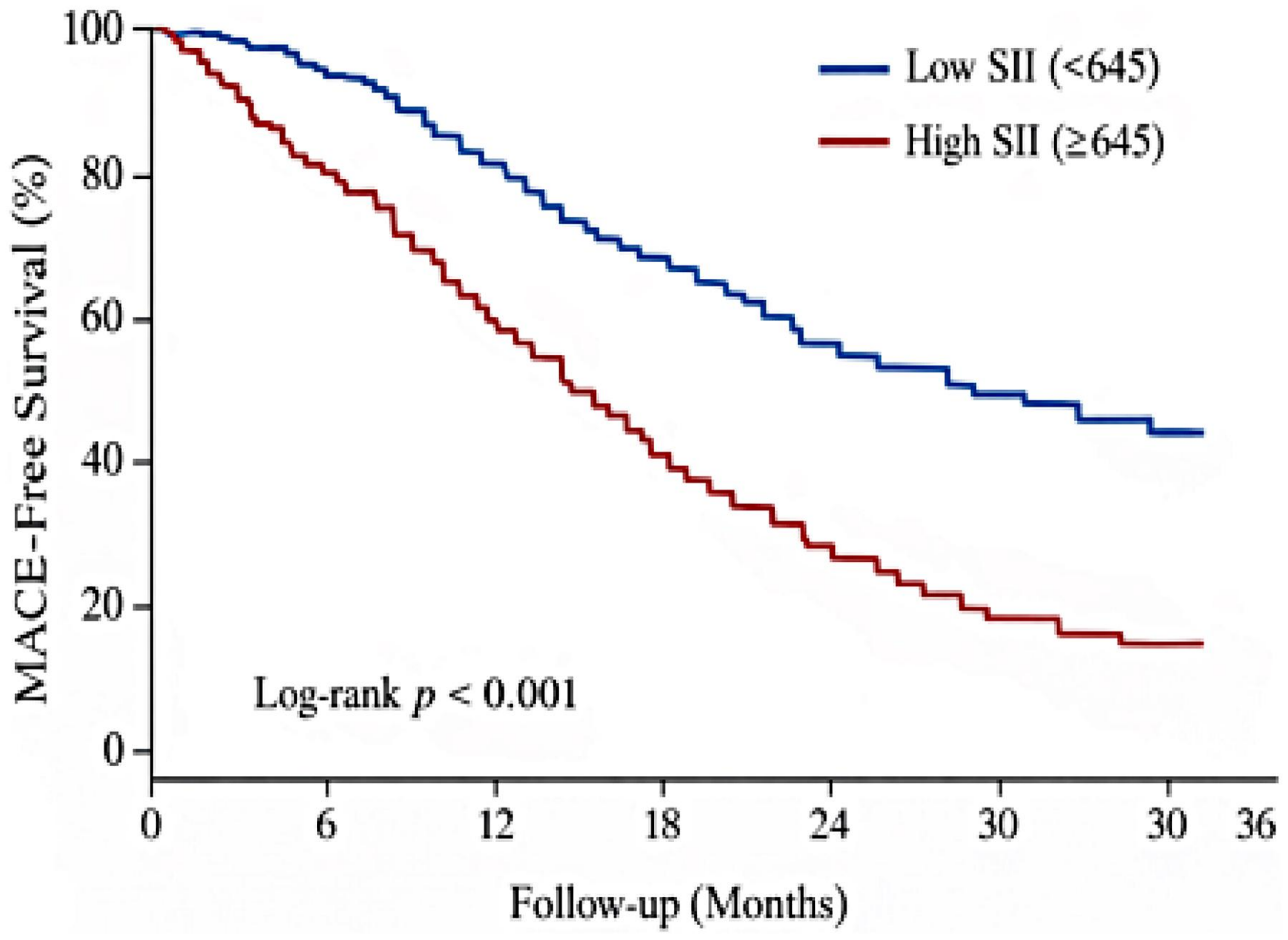
Kaplan–meier analysis of Major adverse cardiovascular event-free survival according to systemic immune-inflammation Index. Kaplan–Meier survival curves showing freedom from major adverse cardiovascular events (MACE) among patients with coronary artery ectasia stratified according to the receiver operating characteristic (ROC)-derived systemic immune-inflammation index (SII) cutoff value of 645. Patients with elevated SII (≥645) demonstrated significantly lower event-free survival compared with those with lower SII (<645) throughout follow-up. Statistical comparison between groups was performed using the log-rank test (*p* < 0.001).

Patients were stratified into two groups according to the ROC-derived SII cut-off value of 645: a high SII group (≥645) and a low SII group (<645). During follow-up, the incidence of major adverse cardiovascular events (MACE) was significantly higher in patients with elevated SII levels. Kaplan–Meier survival analysis demonstrated a marked separation of event-free survival curves between the two groups. As shown in Figure 2, patients in the high SII group exhibited significantly lower MACE-free survival compared with those in the low SII group. At 36 months, the estimated MACE-free survival rate was approximately 41% in the high SII group, compared with 75% in the low SII group.

The difference between survival curves was statistically significant (log-rank *p* < 0.001), indicating that elevated SII is strongly associated with increased risk of adverse cardiovascular outcomes in patients with coronary artery ectasia.

### 3.4 Cox regression analysis

#### 3.4.1 Univariate analysis

Univariate Cox proportional hazards regression analysis was performed to identify potential predictors of major adverse cardiovascular events (MACE) in patients with coronary artery ectasia. The variables evaluated included age, sex, diabetes mellitus, hypertension, current smoking status, low-density lipoprotein cholesterol (LDL-C), high-density lipoprotein cholesterol (HDL-C), left ventricular ejection fraction (LVEF), C-reactive protein (CRP), neutrophil count, lymphocyte count, platelet count, neutrophil-to-lymphocyte ratio (NLR), platelet-to-lymphocyte ratio (PLR), systemic immune-inflammation index (SII), and Markis classification. Variables demonstrating statistical significance in univariate analysis or considered clinically relevant based on prior evidence were subsequently entered into the multivariate Cox regression model. Elevated SII was significantly associated with increased risk of MACE. When analyzed as a continuous variable (per 100-unit increase), SII was associated with a 9% increase in risk (HR 1.09, 95% CI 1.06–1.12, *p* < 0.001). Age was also significantly associated with adverse outcomes (HR 1.04 per year increase, 95% CI 1.01–1.07, *p* = 0.01). Diabetes mellitus conferred a higher risk of events (HR 1.88, 95% CI 1.03–3.44, *p* = 0.04).

Left ventricular ejection fraction (LVEF) was inversely associated with MACE, indicating a protective effect (HR 0.94 per 1% increase, 95% CI 0.91–0.98, *p* = 0.003). Similarly, higher CRP levels were significantly associated with increased risk (HR 1.15 per 1 mg/L increase, 95% CI 1.08–1.23, *p* < 0.001). Advanced ectasia severity (Markis type I–II vs. III–IV) was also associated with adverse outcomes (HR 1.97, 95% CI 1.09–3.55, *p* = 0.02). The results of the univariate Cox regression analysis are summarized in Table 2.

**Table 2:** Univariate Cox regression analysis for MACE.

Variable	Hazard Ratio (HR)	95% Confidence Interval	*p*-value
SII (per 100-unit increase)	1.09	1.06–1.12	<0.001
Age (per year)	1.04	1.01–1.07	0.01
Diabetes mellitus	1.88	1.03–3.44	0.04
LVEF (per 1% increase)	0.94	0.91–0.98	0.003
CRP (per 1 mg/L increase)	1.15	1.08–1.23	<0.001
Markis Type I–II	1.97	1.09–3.55	0.02

#### 3.4.2 Multivariate analysis

To determine whether SII independently predicted major adverse cardiovascular events (MACE), a multivariate Cox proportional hazards regression model was constructed. Variables with *p* < 0.10 in univariate analysis were selected for further evaluation to minimize the risk of excluding potentially important predictors that might demonstrate independent prognostic value after adjustment for confounding factors. In addition, clinically relevant covariates identified from previous cardiovascular literature were considered during model construction. Accordingly, age, diabetes mellitus, left ventricular ejection fraction (LVEF), C-reactive protein (CRP), Markis classification, and SII were entered into the fully adjusted model. When analyzed as a continuous variable [per standard deviation (SD) increase], SII remained a strong independent predictor of MACE. Each 1-SD increase in SII was associated with a 1.72-fold higher risk of adverse events (adjusted HR 1.72, 95% CI 1.34–2.21, *p* < 0.001), even after adjustment for traditional cardiovascular risk factors and inflammatory markers.

When SII was entered as a categorical variable based on the ROC-derived cut-off value of 645, patients in the high SII group (≥645) exhibited a significantly increased risk of MACE compared with those in the low SII group (<645) (adjusted HR 2.48, 95% CI 1.29–4.77, *p* = 0.006).

Among the covariates included in the multivariate model, reduced LVEF remained independently associated with adverse outcomes (adjusted HR 0.95 per 1% increase, 95% CI 0.92–0.99, *p* = 0.01). Diabetes mellitus was also identified as an independent predictor of MACE (adjusted HR 1.79, 95% CI 1.01–3.18, *p* = 0.047). Although age, CRP, and Markis classification were significantly associated with MACE in the univariate analysis, these variables did not retain independent significance after multivariable adjustment. These findings suggest that the prognostic effect of age may be partially mediated through other clinical and inflammatory factors included in the model. These findings indicate that SII provides prognostic information beyond conventional clinical and inflammatory parameters in patients with coronary artery ectasia (Table 3).

**Table 3:** Multivariate Cox regression analysis for MACE.

Variable	Adjusted HR	95% Confidence Interval	*p*-value
SII (per SD increase)	1.72	1.34–2.21	<0.001
High SII (≥645)	2.48	1.29–4.77	0.006
Age (per year)	1.02	0.99–1.05	0.12
Diabetes mellitus	1.79	1.01–3.18	0.047
LVEF (per 1% increase)	0.95	0.92–0.99	0.01
CRP (per 1 mg/L increase)	1.05	0.98–1.13	0.15
Markis Type I–II	1.41	0.77–2.58	0.26

### 3.5 Incremental predictive value of SII

To further evaluate the clinical utility of SII, we assessed whether the addition of SII improved the predictive performance of a baseline model including traditional risk factors (age, diabetes mellitus, LVEF, CRP, and Markis classification). The baseline model demonstrated moderate discriminatory ability for predicting MACE, with a C-statistic of 0.72 (95% CI: 0.64–0.80). After adding SII (as a continuous variable per SD increase) to the model, the C-statistic significantly improved to 0.83 (95% CI: 0.76–0.89), indicating enhanced discrimination. Furthermore, the addition of SII resulted in significant reclassification improvement. The net reclassification improvement (NRI) was 0.31 (95% CI: 0.12–0.49, *p* = 0.002), and the integrated discrimination improvement (IDI) was 0.07 (95% CI: 0.03–0.11, *p* < 0.001). These findings indicate that SII meaningfully improves risk stratification beyond conventional clinical variables (Table 4).

**Table 4:** Comparison of predictive models with and without SII.

Model	C-statistic (95% CI)	NRI (95% CI)	*p*-value	IDI (95% CI)	*p*-value
Baseline Model*	0.72 (0.64–0.80)	—	—	—	—
Baseline + SII	0.83 (0.76–0.89)	0.31 (0.12–0.49)	0.002	0.07 (0.03–0.11)	<0.001

* Baseline model includes age, diabetes mellitus, LVEF, CRP, and Markis classification.

Overall, incorporation of SII into the predictive model enhanced both discrimination and risk reclassification, supporting its incremental prognostic value in patients with coronary artery ectasia. Taken together with the ROC and survival analyses, these findings indicate that SII may serve as a clinically useful biomarker for risk stratification in patients with CAE, providing meaningful prognostic information beyond conventional clinical and inflammatory risk factors.

## 4 Discussion

In this retrospective cohort study of patients with angiographically confirmed coronary artery ectasia, we demonstrated that the systemic immune-inflammation index (SII) is a strong and independent predictor of major adverse cardiovascular events (MACE). Patients who developed adverse events exhibited significantly higher baseline SII levels, and SII showed good discriminatory performance for predicting MACE. Importantly, SII remained independently associated with outcomes after adjustment for conventional cardiovascular risk factors, inflammatory markers, and ectasia severity. Furthermore, incorporation of SII into a baseline predictive model significantly improved discrimination and risk reclassification. These findings suggest that SII provides incremental prognostic information and may serve as a practical risk stratification tool in patients with CAE. Notably, the clinical utility of SII is supported not only by its discriminatory performance on ROC analysis but also by its ability to significantly improve model discrimination and patient risk reclassification when added to conventional clinical predictors.

Coronary artery ectasia is increasingly recognized as a clinically relevant entity rather than a benign anatomical variant. Although often associated with atherosclerosis, CAE is characterized by abnormal arterial remodeling, medial layer degradation, and chronic vascular inflammation (19). Hemodynamic alterations in ectatic segments, including turbulent flow and blood stasis, predispose to thrombus formation and distal embolization, thereby increasing the risk of recurrent ischemic events even in the absence of critical stenosis (2). However, despite these pathophysiological insights, reliable biomarkers for long-term risk assessment in CAE remain limited.

Inflammation has been established as a central driver of atherosclerosis progression and plaque instability. Neutrophils promote endothelial dysfunction and oxidative stress, platelets amplify thrombo-inflammatory signaling, and lymphocyte depletion reflects impaired immune regulation (8, 20). The SII integrates these three components into a single composite index, thereby capturing the balance between pro-inflammatory, pro-thrombotic, and immunoregulatory pathways. Compared with isolated markers such as neutrophil-to-lymphocyte ratio (NLR) or platelet-to-lymphocyte ratio (PLR), SII provides a more comprehensive assessment of systemic inflammatory burden.

Previous studies have consistently demonstrated that elevated SII is associated with increased mortality and recurrent cardiovascular events across various cardiovascular settings, including patients undergoing percutaneous coronary intervention and those with acute coronary syndromes (13, 21, 22). However, data regarding SII in CAE have been limited primarily to cross-sectional analyses evaluating its association with the presence or angiographic severity of ectasia rather than clinical outcomes.

Tosu and Biter reported that SII levels were significantly higher in patients with isolated CAE compared with individuals with normal coronary arteries, suggesting an inflammatory contribution to ectatic remodeling (16). Karakayali et al. and Esenboğa et al. further demonstrated that SII was associated with the presence of isolated CAE independent of traditional risk factors (17, 23). More recently, Dindas et al. and Candemir et al. showed that SII correlated with the anatomical severity of CAE according to the Markis classification (18, 24). While these studies established a link between systemic inflammation and ectatic coronary morphology, they did not evaluate long-term adverse cardiovascular outcomes.

The present study extends prior findings by demonstrating that SII is not only associated with the presence or severity of CAE but also independently predicts future cardiovascular events. In our cohort, patients who experienced MACE had markedly elevated SII levels at baseline. Receiver operating characteristic analysis demonstrated strong discriminatory ability, and Kaplan–Meier survival analysis revealed significant separation between high and low SII groups. Importantly, the independent prognostic value of SII persisted after adjustment for age, diabetes mellitus, left ventricular function, CRP levels, and ectasia severity.

The persistence of SII as an independent predictor despite adjustment for CRP is particularly noteworthy. CRP is a well-established inflammatory biomarker in cardiovascular disease; however, it reflects primarily hepatic acute-phase response rather than the integrated interaction between immune cells and thrombosis. The fact that SII retained significance while CRP did not suggests that SII may better capture the pathophysiological processes underlying adverse events in CAE. This finding aligns with prior cardiovascular studies in which SII demonstrated superior prognostic performance compared with single inflammatory markers (21, 22).

Another important observation is the incremental predictive value of SII beyond traditional risk factors. The addition of SII to a baseline model significantly improved the C-statistic and enhanced both net reclassification improvement (NRI) and integrated discrimination improvement (IDI). These findings indicate that SII does not merely correlate with outcomes but meaningfully enhances risk stratification. From a clinical perspective, such improvement in discrimination and reclassification supports the potential integration of SII into routine risk assessment frameworks for patients with CAE.

The mechanistic link between elevated SII and adverse outcomes in CAE is biologically plausible. Ectatic vessels are characterized by abnormal wall stress, endothelial dysfunction, and altered shear forces, which promote inflammatory activation and platelet aggregation (2, 19). Elevated neutrophil counts may reflect enhanced oxidative stress and proteolytic activity within the vascular wall, contributing to further structural instability. Increased platelet counts may facilitate thrombus formation within dilated segments, while reduced lymphocyte counts may indicate impaired immune surveillance and systemic stress. The combination of these alterations, captured by SII, likely reflects a pro-thrombotic and pro-inflammatory milieu that predisposes patients to recurrent ischemic events.

In addition to SII, diabetes mellitus and reduced left ventricular ejection fraction were independently associated with adverse outcomes in our multivariate analysis. These findings are consistent with established literature demonstrating that metabolic dysregulation and impaired ventricular function increase cardiovascular risk (25). Although age was associated with MACE in the univariate analysis, it did not remain statistically significant after adjustment for other clinical and inflammatory variables, suggesting that its prognostic effect may be influenced by coexisting risk factors and inflammatory burden. Notably, both advanced age and diabetes mellitus are closely linked to chronic low-grade systemic inflammation, which may partially explain the elevated SII levels observed among patients who subsequently developed MACE (26). Aging is associated with a phenomenon known as ‘inflammaging,’ characterized by persistent activation of innate immune pathways, increased circulating pro-inflammatory cytokines, endothelial dysfunction, and heightened oxidative stress. These processes promote neutrophil activation and platelet reactivity while impairing adaptive immune regulation, thereby contributing to higher SII values. Similarly, diabetes mellitus is characterized by chronic hyperglycemia-induced oxidative stress, endothelial injury, platelet hyperactivity, and persistent inflammatory activation. These mechanisms can increase neutrophil and platelet counts while reducing lymphocyte-mediated immune homeostasis, resulting in an elevated SII (27). Consequently, the higher prevalence of older age and diabetes among patients experiencing MACE may have contributed to an enhanced inflammatory and thrombotic milieu, thereby increasing susceptibility to adverse cardiovascular events. The persistence of SII as an independent predictor after multivariable adjustment suggests that it captures the cumulative impact of these pathophysiological processes beyond the individual contribution of traditional risk factors (28). Interestingly, although advanced Markis classification was associated with events in univariate analysis, it did not retain independent significance in the fully adjusted model. This observation suggests that systemic inflammatory burden may be a stronger determinant of prognosis than anatomical extent of ectasia alone.

The clinical implications of our findings are noteworthy. Because SII is derived from routine complete blood count parameters, it is inexpensive, readily available, and easily calculated in daily clinical practice. Identification of high-risk CAE patients using SII may allow more intensive follow-up, optimization of antithrombotic strategies, and stricter control of modifiable risk factors. Although prospective validation is warranted, incorporation of SII into risk assessment algorithms may help address the current gap in prognostic stratification for CAE.

### 4.1 Study limitations

This study has several limitations that should be considered when interpreting the findings. First, this was a retrospective single-center study, which may introduce selection bias and limit the generalizability of the findings. Because all patients were recruited from a single tertiary medical center, the study population may not fully represent the broader spectrum of patients with coronary artery ectasia encountered in different geographic regions, healthcare systems, or clinical practice settings. Furthermore, local referral patterns, treatment strategies, and patient characteristics may have influenced the observed associations. Therefore, caution should be exercised when extrapolating these findings to other populations. Second, SII was calculated from a single baseline measurement, and dynamic changes in inflammatory status during follow-up were not assessed. Third, although multivariate adjustment was performed, residual confounding and unmeasured variables cannot be entirely excluded due to the observational nature of the study. In particular, detailed information regarding medical therapies during follow-up, including antiplatelet agents, statins, anticoagulants, and other cardiovascular medications, was not consistently available for all patients. These treatments may influence both inflammatory status and cardiovascular outcomes and therefore could have affected the observed associations. Additionally, variations in medication adherence, treatment intensity, and therapeutic modifications over time were not captured in the present analysis. Consequently, the potential confounding effect of medical management cannot be completely excluded. Finally, the sample size was relatively modest, particularly with respect to the number of observed MACE events (*n* = 36). Although statistically significant associations were identified, the limited number of events may have reduced statistical power and affected the stability and precision of multivariable risk estimates. In addition, the optimal SII cutoff value identified in the present study was derived from the study cohort using ROC curve analysis and has not been externally validated. Therefore, the generalizability and clinical applicability of this threshold to other patient populations remain uncertain. Independent validation in larger multicenter cohorts is necessary before widespread clinical implementation can be recommended. In studies with relatively few outcome events, there is an increased possibility of model overfitting and wider confidence intervals, which may influence the reliability of effect estimates. Therefore, the findings should be interpreted with appropriate caution. Nevertheless, the consistent association between elevated SII and adverse outcomes across ROC, survival, and multivariate analyses supports the robustness of the observed relationship. Future large-scale prospective multicenter studies with a greater number of clinical events are required to validate these findings, improve statistical precision, and further establish the prognostic utility of SII in patients with coronary artery ectasia.

Despite these limitations, this study provides novel evidence that SII independently predicts major adverse cardiovascular events in patients with coronary artery ectasia and offers incremental prognostic value beyond traditional risk factors, further highlighting the importance of systemic inflammation in the clinical course of CAE. By demonstrating both strong discriminatory performance and incremental prognostic value beyond traditional risk factors, our findings highlight the importance of systemic inflammation in the pathophysiology and clinical course of CAE. Future prospective investigations are warranted to validate these results and to explore whether targeted anti-inflammatory or antithrombotic strategies guided by SII may improve outcomes in this high-risk population.

## 5 Conclusion

In this retrospective cohort study of patients with angiographically confirmed coronary artery ectasia, the systemic immune-inflammation index emerged as a strong and independent predictor of major adverse cardiovascular events. Elevated baseline SII levels were significantly associated with increased risk of cardiovascular death, myocardial infarction, stroke, and target vessel revascularization during follow-up. Importantly, the prognostic value of SII remained robust after adjustment for conventional cardiovascular risk factors, inflammatory markers, and angiographic severity of ectasia. Beyond its independent association with adverse outcomes, SII demonstrated meaningful incremental predictive value. The addition of SII to a baseline clinical model significantly improved discrimination and risk reclassification, underscoring its potential utility in clinical practice. Given that SII is derived from routinely available hematologic parameters, it represents a simple, cost-effective, and easily accessible biomarker for risk stratification in patients with CAE. These findings highlight the central role of systemic inflammation in the pathophysiology and prognosis of coronary artery ectasia. Prospective multicenter studies involving larger and more diverse patient populations are warranted to validate these findings, assess their generalizability, and determine whether SII-guided management strategies may improve long-term outcomes in this patient population.

## Data Availability

The raw data supporting the conclusions of this article will be made available by the authors, without undue reservation.

## References

[1] ElGuindy MS ElGuindy AM. Aneurysmal coronary artery disease: an overview. Glob Cardiol Sci Pract. (2017) 2017(3):e201726. 10.21542/gcsp.2017.26 29564347 PMC5856968

[2] Manginas A Cokkinos DV. Coronary artery ectasias: imaging, functional assessment and clinical implications. Eur Heart J. (2006) 27(9):1026–31. 10.1093/eurheartj/ehi725 16415301

[3] Liu R Zhao H Gao X Liang S. Is coronary artery ectasia a progressive disease? A self-controlled retrospective cohort study. Front Cardiovasc Med. (2021) 8:774597. 10.3389/fcvm.2021.774597 34938789 PMC8685394

[4] Wozniak P Iwanczyk S Blaszyk M Stepien K Lesiak M Mularek-Kubzdela T. Coronary artery aneurysm or ectasia as a form of coronary artery remodeling: etiology, pathogenesis, diagnostics, complications, and treatment. Biomedicines. (2024) 12(9):1984. 10.3390/biomedicines12091984 39335497 PMC11428638

[5] Baysal SS Koc S Gunes A Altiparmak IH. Endothelium biomarkers endocan and thrombomodulin levels in isolated coronary artery ectasia. Eur Rev Med Pharmacol Sci. (2018) 22(14):4677–82. 10.26355/eurrev_201807_15528 30058709

[6] Giucă A Rocsoreanu A Şerban M Roşca M Iancu M Carp A. Isolated coronary artery ectasia presenting as Inferior-posterior stemi—a case-based state-of-the-art review of the current literature. Romanian Journal of Cardiology. (2023) 33(4):147–60. 10.2478/rjc-2023-0025

[7] Doi T Kataoka Y Noguchi T Shibata T Nakashima T Kawakami S. Coronary artery ectasia predicts future cardiac events in patients with acute myocardial infarction. Arterioscler Thromb Vasc Biol. (2017) 37(12):2350–5. 10.1161/ATVBAHA.117.309683 29051141

[8] Ajoolabady A Pratico D Lin L Mantzoros CS Bahijri S Tuomilehto J. Inflammation in atherosclerosis: pathophysiology and mechanisms. Cell Death Dis. (2024) 15(11):817. 10.1038/s41419-024-07166-8 39528464 PMC11555284

[9] Zhang X Kang Z Yin D Gao J. Role of neutrophils in different stages of atherosclerosis. Innate Immun. (2023) 29(6):97–109. 10.1177/17534259231189195 37491844 PMC10468622

[10] Voronkov NS Maslov LN Vyshlov EV Mukhomedzyanov AV Ryabov VV Derkachev IA. Do platelets protect the heart against ischemia/reperfusion injury or exacerbate cardiac ischemia/reperfusion injury? The role of pdgf, vegf, and paf. Life Sci. (2024) 347:122617. 10.1016/j.lfs.2024.122617 38608835

[11] Olasinska-Wisniewska A Urbanowicz T Perek B Misterski M Grodecki K Grygier M. Predictive value of monocyte-to-lymphocyte ratio in differentiating heart failure with reduced ejection fraction in patients with severe aortic stenosis-a retrospective analysis. J Clin Med. (2024) 13(20):6249. 10.3390/jcm13206249 39458199 PMC11508807

[12] Tsilimigras DI Moris D Mehta R Paredes AZ Sahara K Guglielmi A. The systemic immune-inflammation Index predicts prognosis in intrahepatic cholangiocarcinoma: an international multi-institutional analysis. HPB (Oxford). (2020) 22(12):1667–74. 10.1016/j.hpb.2020.03.011 32265108

[13] Zhao J Lv H Yin D Zhou X Zhu H Guo L. Systemic immune-inflammation Index predicts long-term outcomes in patients with three-vessel coronary disease after revascularization: results from a large cohort of 3561 patients. J Inflamm Res. (2022) 15:5283–92. 10.2147/JIR.S385990 36120186 PMC9480584

[14] Marchi F Pylypiv N Parlanti A Storti S Gaggini M Paradossi U. Systemic immune-inflammation Index and systemic inflammatory response Index as predictors of mortality in st-elevation myocardial infarction. J Clin Med. (2024) 13(5):1256. 10.3390/jcm13051256 38592104 PMC10931789

[15] Su G Zhang Y Xiao R Zhang T Gong B. Systemic immune-inflammation Index as a promising predictor of mortality in patients with acute coronary syndrome: a real-world study. J Int Med Res. (2021) 49(5):3000605211016274. 10.1177/03000605211016274 34034539 PMC8161892

[16] Tosu AR Biter HI. Association of systemic immune-inflammation Index (sii) with presence of isolated coronary artery ectasia. Arch Med Sci Atheroscler Dis. (2021) 6:e152–7. 10.5114/amsad.2021.109253 34703943 PMC8525247

[17] Esenboga K Kurtul A Yamanturk YY Akbulut IM Tutar DE. Comparison of systemic immune-inflammation Index levels in patients with isolated coronary artery ectasia versus patients with obstructive coronary artery disease and normal coronary angiogram. Scand J Clin Lab Invest. (2022) 82(2):132–7. 10.1080/00365513.2022.2034034 35143364

[18] Dindas F Koyun E Turkyilmaz E Abacioglu OO Yildirim A Sahin A. Systemic immune inflammation Index is a novel marker in predicting the presence and severity of isolated coronary artery ectasia. Arq Bras Cardiol. (2023) 120(1):e20220056. 10.36660/abc.20220056 36629598 PMC9833212

[19] Antoniadis AP Chatzizisis YS Giannoglou GD. Pathogenetic mechanisms of coronary ectasia. Int J Cardiol. (2008) 130(3):335–43. 10.1016/j.ijcard.2008.05.071 18694609

[20] Irwandi RA Chiesa ST Hajishengallis G Papayannopoulos V Deanfield JE D'Aiuto F. The roles of neutrophils linking periodontitis and atherosclerotic cardiovascular diseases. Front Immunol. (2022) 13:915081. 10.3389/fimmu.2022.915081 35874771 PMC9300828

[21] Saylik F Akbulut T. Systemic immune-inflammation Index predicts Major cardiovascular adverse events in patients with st-segment elevated myocardial infarction. Arq Bras Cardiol. (2022) 119(1):14–22. 10.36660/abc.20210412 35830117 PMC9352114

[22] Wang S Zhang G. Association between systemic immune-inflammation Index and adverse outcomes in patients with acute coronary syndrome: a meta-analysis. Angiology. (2025) 76(10):946–54. 10.1177/00033197241263399 38904183

[23] Karakayali M Altunova M Yakisan T Aslan S Omar T Artac I. The relationship between the systemic immune-inflammation Index and ischemia with non-obstructive coronary arteries in patients undergoing coronary angiography. Arq Bras Cardiol. (2024) 121(2):e20230540. 10.36660/abc.20230540 38597536 PMC12092018

[24] Candemir M Kiziltunc E Nurkoc S Sahinarslan A. Relationship between systemic immune-inflammation Index (sii) and the severity of stable coronary artery disease. Angiology. (2021) 72(6):575–81. 10.1177/0003319720987743 33685239

[25] Eckel RH Bornfeldt KE Goldberg IJ. Cardiovascular disease in diabetes, beyond glucose. Cell Metab. (2021) 33(8):1519–45. 10.1016/j.cmet.2021.07.001 34289375 PMC8411849

[26] Zhu S Li H Ou Z Zheng M Yuan W. Systemic immune-inflammation Index outperforms conventional inflammatory markers in predicting cardiovascular outcomes in heart failure with preserved ejection fraction. J Inflamm Res. (2026) 19:1–11. 10.2147/JIR.S568473 PMC13003773 41867448

[27] García-Domínguez M. Pathological and inflammatory consequences of aging. Biomolecules. (2025) 15(3):404. 10.3390/biom15030404 40149940 PMC11939965

[28] González-Juanatey C Anguita-Sánchez M Barrios V Núñez-Gil I Gómez-Doblas JJ García-Moll X. Impact of advanced age on the incidence of major adverse cardiovascular events in patients with type 2 diabetes mellitus and stable coronary artery disease in a real-world setting in Spain. J Clin Med. (2023) 12(16):5218. 10.3390/jcm12165218 37629262 PMC10456002

